# Phylogenetic relationships in genus *Arachis *based on ITS and 5.8S rDNA sequences

**DOI:** 10.1186/1471-2229-10-255

**Published:** 2010-11-19

**Authors:** Marcelo D Bechara, Márcio C Moretzsohn, Darío A Palmieri, Jomar P Monteiro, Maurício Bacci, Joaquim Martins, José FM Valls, Catalina R Lopes, Marcos A Gimenes

**Affiliations:** 1 Universidade de Marília, UNIMAR - Marília, SP, Brazil; 2 Embrapa Recursos Genéticos e Biotecnologia, C.P. 02372, 70770-917, Brasília, DF, Brazil; 3 Departamento de Ciências Biológicas, Faculdade de Ciências e Letras, UNESP - Universidade Estadual Paulista, 19806-900, Assis, SP, Brazil; 4 Departamento de Genética, Instituto de Biociências, UNESP - Universidade Estadual Paulista, 18618-000, Botucatu, SP, Brazil; 5 Departamento de Bioquímica e Microbiologia, Instituto de Biociências, UNESP - Universidade Estadual Paulista, 13506-900, Rio Claro, SP, Brazil; 6 Centro de Estudos de Insetos Sociais, Instituto de Biociências, UNESP - Universidade Estadual Paulista, 13506-900, Rio Claro, SP, Brasil

## Abstract

**Background:**

The genus *Arachis *comprises 80 species and it is subdivided into nine taxonomic sections (*Arachis*, *Caulorrhizae*, *Erectoides*, *Extranervosae*, *Heteranthae*, *Procumbentes*, *Rhizomatosae*, *Trierectoides*, and *Triseminatae*). This genus is naturally confined to South America and most of its species are native to Brazil. In order to provide a better understanding of the evolution of the genus, we reconstructed the phylogeny of 45 species using the variation observed on nucleotide sequences in internal transcribed spacer regions (ITS1 and ITS2) and 5.8 S of nuclear ribosomal DNA.

**Results:**

Intraspecific variation was detected, but in general it was not enough to place accessions of the same species in different clades. Our data support the view that *Arachis *is a monophyletic group and suggested *Heteranthae *as the most primitive section of genus *Arachis*. The results confirmed the circumscriptions of some sections (*Caulorrhizae*, *Extranervosae*), but raised questions about others. Sections *Erectoides*, *Trierectoides *and *Procumbentes *were not well defined, while sections *Arachis *and *Rhizomatosae *seem to include species that could be moved to different sections. The division of section *Arachis *into A and B genome species was also observed in the phylogenetic tree and these two groups of species may not have a monophyletic origin. The 2n = 2x = 18 species of section *Arachis *(*A. praecox*, *A*. *palustris *and *A. decora*) were all placed in the same clade, indicating they are closely related to each other, and their genomes are more related to B genome than to the A genome. Data also allowed insights on the origin of tetraploid *A. glabrata*, suggesting rhizome appeared twice within the genus and raising questions about the placement of that species in section *Rhizomatosae*.

**Conclusion:**

The main clades established in this study in general agreed with many other studies that have used other types of evidences and sets of species, being some of them included in our study and some not. Thus, the relationships established can be a useful framework for future systematic reviews of genus *Arachis *and for the selection of species to pre-breeding programs.

## Background

The genus Arachis originated in South America, where all the cultivated and wild species are found, and includes 80 described species [1,2]. Groundnut, the allotetraploid species *A*. *hypogaea *(genome formula AABB), is the most important species of the genus because it is cultivated as an oilseed crop and as a direct source of human food. The genus also includes species such as *A. glabrata *(section *Rhizomatosae*) and *A. pintoi *(section *Caulorrhizae*), which are frequently used in cultivated pastures.

Many studies have focused on the taxonomy of genus *Arachis *(Table 1). The first published classification divided the genus into six sections, some of which were sub-divided in series [3]. A taxonomic revision of this genus resulted in the inclusion of section *Triseminalae *[4]. Krapovickas [5] divided the genus into eight sections and classified one of the series of section *Erectoides*, established by Gregory *et al*. [4], as a new section called *Procumbensae*. The genus is currently divided into nine sections (*Arachis*, *Caulorrhizae*, *Erectoides*, *Extranervosae*, *Heteranthae*, *Procumbentes*, *Rhizomatosae*, *Trierectoides*, and *Triseminatae*) based on morphology, geographical distribution and crossability [1]. In this last revision, two species with trifoliolate leaves from section *Erectoides *were transferred to a new section called *Trierectoides*.

**Table 1:** Classifications of genus Arachis*.

Krapovickas (1969)	Krapovickas (1975)	**Gregory et al**. (1973, 1980)	Krapovickas (1990)	Krapovickas and Gregory (1994)	Number of described species	Number of species analyzed	Genomes
AXONOMORPHAE	ARACHIS	AXONOMORPHAE	ARACHIS	ARACHIS	31	25	A,B,D,F,K, AB
		Annuae	Annuae				
		Perennes	Perennes				
		Amphiploides	Amphiploides				
ERECTOIDES	TRIERECTOIDES	ERECTOIDES	ERECTOIDES	ERECTOIDES	14	4	E
		Trifoliolatae	Tetrafoliolate				
	TETRAERECTOIDES	Tetrafoliolate	Trifoliolatae	TRIERECTOIDES	2	2	E
		Procumbensae	PROCUMBENSAE	PROCUMBENTES	10	5	E
CAULORRHIZAE	CAULORRHIZAE	CAULORRHIZAE	CAULORRHIZAE	CAULORRHIZAE	2	2	C
RHIZOMATOSAE	RHIZOMATOSAE	RHIZOMATOSAE	RHIZOMATOSAE	RHIZOMATOSAE	2	2	R
		Prorhizomatosae	Prorhizomatosae	Prorhizomatosae			
		Eurhizomatosae	Eurhizomatosae	Eurhizomatosae			
EXTRANERVOSAE	EXTRANERVOSAE	EXTRANERVOSAE	EXTRANERVOSAE	EXTRANERVOSAE	10	3	Ex
AMBINERVOSAE	AMBINERVOSAE	PSEUDAXONOMORPHAE	AMBINERVOSAE	HETERANTHAE	6	1	Am
		TRISEMINALAE	TRISEMINALAE	TRISEMINATAE	1	1	T

*Modified from Valls and Simpson [69].

The systematic relationships among *Arachis *species have been inferred using different molecular markers, such as RAPDs [6], storage proteins [7,8], isozymes [9,10], variation on sequence of desaturase genes [11], RFLP [12], microsatellites [13-16], AFLPs [17], cytogenetic and molecular data from AFLP and the *trn*T-F plastid region [18], FISH and GISH [19-21]. However, most of these studies included only species belonging to section *Arachis*. Just recently one study that included species from all sections was published [22].

Understanding the phylogenetic relationships among *Arachis *species would contribute to the systematics of the genus, comprehension of the origins and evolution of species and sections and the use of species of genus *Arachis*. For instance, the circumscriptions of some sections are based in criterions that may not reflect phylogenetic relationships. The maintenance of species associated respectively to the A and the B genomes of the cultivated peanut in a single section does not seem to be natural and it may be an artificial construction derived from the existence of the peanut, a fixed amphidiploid gathering genetic material from species that when crossed to each other produce unfertile hybrids at the diploid level. Also, section *Rhizomatosae*, a very important group from the standpoint of forage production, comprises polyploid species (*A. glabrata*, *A. pseudovillosa, A. nitida*) and one diploid (*A. burkartii*) that RAPD [23] and microsatellite data [24] showed to be very distinct from the other species in this section. As mentioned before, phylogenetic information will also have great impact in the utilization of the species, mainly those from sections that comprise cultivated species. For instance, for many years *A. batizocoi *was considered the donor of the B genome of *A. hypogaea *and that species was used with moderated success in pre-breeding programs. However, molecular and cytogenetic evidences showed that *A. ipaënsis *was the most probable donor of B genome of *A. hypogaea *and *A. duranensis *the donor of the A genome [25]. That information was corroborated based on molecular cytogenetics [19] and crossability [26]. The number of accessions of *A*. *duranensis *is very large [1] comprising large variability that could be used to improve *A. hypogaea *through introgression into its A genome. Besides, the relationships between A genome species are well defined and they show very good crossability to each other [1]. On the other hand, there is an unique accession of *A. ipaënsis *available and the relationships among species of section *Arachis *that do not have the A genome is not well defined. The non A genome species group is very diverse comprising species with different degrees of affinity to the B genome of *A. hypogaea *[18,27]. Phylogeny and genomic data also allow a better understanding of the evolution in section *Arachis*. For instance, recently the first comparative genomic study between the genomes of *A. hypogaea *using microsatellite markers and two map populations resulting from crosses between two A genome species and two B genome species was published [28]. The comparison between the B genome and A genome maps revealed a high degree of synteny. The development of genetic maps for *Arachis *diploid wild species with A and B genomes associated to phylogenetic studies effectively is a significant advance towards the construction of a transferable reference map for *Arachis*.

The DNA sequence variation observed in the internal transcribed spacers (ITS1 and ITS2) of nuclear rDNA, located between the 18 S and 26 S rDNA coding regions, has been largely used for phylogenetic analysis at plant genus and species discrimination levels [29-34]. The sequences are relatively easy to align because few length variations have been observed at the genus level in flowering plants, they are long enough to offer a sufficient number of potential characters for phylogenetic reconstruction, and are flanked by regions that are highly conserved within genera, thus simplifying the isolation and sequencing of the region through the use of universal primers [35].

The objective of this work was to establish the phylogenetic relationships among species of the genus *Arachis*. The polymorphism in sequences of internal transcribed spacers ITS1 and ITS2 and 5.8 S rDNA coding region was used to determine relationships among 45 species of genus *Arachis*.

## Results and Discussion

We have analyzed 55 accessions encompassing 45 *Arachis *species and the nine taxonomical sections. Consensus sequences were obtained for each accession using four to ten reads. The number of reads per accession varied because their quality also varied. For some species only four reads were necessary to get a good quality consensus sequence and for some ten reads were necessary.

In spite of the large number of studies using ITS to infer phylogeny in a very large number of genus and families, some authors have criticized the use of these genomic regions. ITS data may cause incongruence due to the various mechanisms that can influence ITS variation [33]. Among the most prevalent complications for phylogenetic inference is the existence in many plant genomes of extensive sequence variation, arising from ancient or recent array duplication events, genomic harboring of pseudogenes in various states of decay, and/or incomplete intra- or interarray homogenization [32]. Despite that, we have used ITS region to infer phylogeny in genus *Arachis *and, to have insights in how much the variation in this region may have interfered in the species relationship establishment, we included more than one individual from six species (*A. pintoi*: two accessions; *A. major*: two accessions; *A. paraguariensis*: two accessions; *A. magna*: two accessions, *A*. *kuhlmannii*: four accessions, *A. hoehnei*: three accessions). We also included species that are very related to each other based on different types of evidences, such as *A*. *hypogaea *and *A. monticola *[12,16,36] and *A. repens *and *A. pintoi *[37,38] and included groups of species whose high affinity was demonstrated by many different methods, such as the A genome species [21].

**Figure 1:**
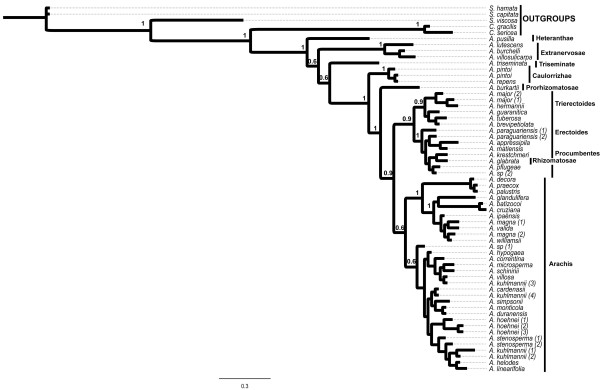
**Phylogenetic tree obtained from Bayesian Inference Analysis**. Numbers ahead nodes are the *posterior *probabilities support values. Three branchs are proportional to mutational events sampled in sequences alignment.

As it can be seen in Figure 1, there was variation between accessions of the same species which may have been caused by the ITS variation cited above. The variation found for three species (*A. pintoi*, *A. major*, *A. hoehnei*) was not enough to place their accessions away from each other. *Arachis magna *accessions were not placed together but they were in the same clade. *Arachis paraguariensis *and *A. kuhlmannii *accessions were, in general, placed away from each other.

The two accessions of *A. paraguariensis *analyzed belong to different subspecies (*A. paraguariensis *subsp. *paraguariensis *and *A. paraguariensis *subsp. *capibarensis*). The differentiation between these two subspecies based on morphology is very difficult but they are considered as different subspecific taxa based on their distinct geographic distribution [1] and SAT chromosome morphology [39].

Four accessions of *A. kuhlmannii *were analyzed (VPoBi 9375 - *A. kuhlmannii *1; VSGr 6404 - *A. kuhlmannii *- 2; VPzRcSgSv 13530 - *A. kuhlmannii *3; VSPmSv 13721 - *A. kuhlmannii *4). All of them were placed in the A genome clade, but into three different subclades. *Arachis kuhlmannii *1 and 2 were placed close to each other in a clade that also included *A. helodes *and *A. linearifolia*. *Arachis kuhlmannii *3 was placed in a subclade with *A. hypogaea*, *A. correntina*, *A. microsperma, A. schininii *and *A. villosa*, while *A. kuhlmannii* 4 in a clade with *A. cardenasii*, *A. simpsonii*, *A. monticola *and *A. duranensis*. Accessions of *A. kulhmannii *were also placed in different subclusters based on microsatellite and AFLP data [16,17]. In a dendrogram obtained using RAPD data, *A. kuhlmannii *1, 2 and 4 grouped together and *A. kuhlmannii *3 was placed in a different group that comprised accessions from Mato Grosso do Sul State in Brazil [40]. *Arachis kuhlmannii *4 was collected in Brazil near the frontier with Bolivia, and probably is more distinct from the other *A. kuhlmannii *accessions and more related to *A. cardenasii*. In the RAPD dendrogram, *A. kuhlmannii *4 grouped to *A. simpsonii *accession VSPmSv 13728, which was collected in Bolivia, in the same population where the species typus was collected.

Thus, the placement of *A. paraguariensis *and *A*. *kuhlmannii *accessions may have been due to factors not related exclusively to the variation found in ITS regions since our data corroborates previous studies demonstrating high variability within these species.

The closely related allopolyploid species *A. hypogaea *and *A. monticola *were placed in different clades. It is known that *A. monticola *is the closest related species to *A. hypogaea *[1]. Despite that, *A. monticola *was considered as a distinct species from *A. hypogaea *[1]. However, some doubts about its classification still remain, because this species has a high crossability with *A. hypogaea *[1,41]. It is also an allotetraploid and both species have identical genomes [19,36,39]. Furthermore part of their geographic distribution overlaps [1]. Molecular marker data also confirmed the very close genetic relationship between these two species [6,11,13,15,17,42,43]. In our case, despite the causes of variation in ITS regions, the observed placement of *A. hypogaea *and *A. monticola *was certainly influenced by the fact that these species have ITS regions from A and B genomes which our results showed to be different and seem to be specific for each genome type found in genus *Arachis*. The sequences of *A. hypogaea *and *A. monticola *included in this study represent one of the genomes and may even be a mixture of the sequences from both. As mentioned above the close relationships between these two species have been demonstrated using different evidences. Thus, our data is limited to infer phylogenetic relationships between the allopolyploids as well as their relationships to the wild diploid species.

Section *Heteranthae *showed a basal position, followed by *Extranervosae*, *Triseminatae*, *Caulorrhizae*, and *Rhizomatosae *Ser. *Prorhizomatosae, Procumbentes*/*Trierectoides*/*Erectoides/Rhizomatosae *and *Arachis*. Despite the lack of dating analysis, the position of clades in relation to the outgroups agreed with DNA content analysis showing that species with greater DNA content were included in sections believed to have a more recent origin (*Procumbentes, Caulorrhizae*, *Rhizomatosae *and *Arachis*), whereas those with lower DNA content in the most primitive or ancient sections (*Extranervosae*, *Heteranthae *and *Triseminatae*) [44]. Sections *Extranervosae*, *Heteranthae *and *Triseminatae *are believed to be among the oldest sections in genus *Arachis *based on their affinity with genus *Stylosanthes *[1].

### Section circumscriptions

The results confirmed the circumscriptions of sections *Caulorrhizae *and *Extranervosae *and suggested some sections may not be natural groups. The circumscriptions of sections *Erectoides, Trierectoides *and *Procumbentes *were not well defined, suggesting that these three sections could be grouped in one or two sections. On the other hand, our data suggested sections *Arachis *and *Rhizomatosae *could be divided into two new sections each.

Sections *Trierectoides*, *Erectoides *and *Procumbentes *seem to be a monophyletic group. Species from these sections were distributed in two sub-clades. One of them comprised three species of section *Erectoides *(*A. major*, *A. hermannii *and *A. brevipetiolata*) and two species of section *Trierectoides *(*A. guaranitica *and *A. tuberosa*). *Arachis brevipetiolata *was more related to *Trierectoides *than to the other two species of *Erectoides*. The other sub-clade comprised one species of section *Erectoides *(*A. paraguariensis*), four species of section *Procumbentes *(*A. appressipila, A. kretschmeri, A. matiensis, A. pflugeae*, and *A*. sp. 2) and one species of section *Rhizomatosae *(*A. glabrata*). As can be seen in Table 1, species from sections *Trierectoides*, *Erectoides *and *Procumbentes *were all put together in the same section for many years and after three taxonomic revisions of genus *Arachis*. Only in 1990, some species were put in a new section called *Procumbensae *that is the actual *Procumbentes *section. Our data supported the first classification [3].

Section *Arachis *comprises species of three types of genomes (A, B and D). The crossability and inter-specific hybrid fertility between A genome species and between some B genome species are very high [12,18]. However, crosses between A and B genome species result in unfertile hybrids [1]. The cultivated peanut is fertile just because it had its chromosomes duplicated, having a diploid-like meiosis, with no pairing of chromosomes from different genomes. Thus, based on the use of crossability for the establishment of the taxonomic sections it may be considered that, if the peanut have not evolved, most *Arachis *specialists would certainly assign the A genome and B genome species to distinct sections.

The presence of rhizomes is the main reason for a species to be in section *Rhizomatosae*, which comprises three polyploidy species (*A. glabrata*, *A. pseudovillosa, A. nitida*) and one diploid (*A. burkartii*). RAPD [23] and microsatellite data [24] showed that the diploid species is very distinct from the other species of this section. The different ploidy levels and variation in the ITS could result in the misplacement of *A. glabrata *or *A. burkartii*. However, we observed in section *Arachis*, which also includes diploid and polyploidy species that ITS variation may result in some unexpected placement of species, but that is not enough for their placement in clades of different sections.

Sections *Triseminatae *and *Heteranthae *had just one species analyzed but they were placed in the tree in individual branches suggesting these sections are also natural groups. *Arachis burkartii *had a placement very similar to those species from sections *Triseminatae *and *Heteranthae*. Thus, based on ours and previous data [23,24] the establishment of a new section for *A. burkartii *should be considered.

### Section *Arachis*

The two genomes presented in domesticated peanut differ by one striking feature: one of the genomes (A) has a pair of chromosomes, called A, which is conspicuously smaller than the other chromosomes, while the other genome lacks this small chromosome [25]. The species of section *Arachis *are therefore classified as having A or B genomes based on the presence or absence of the A pair. A third genome was identified in the genus, the D genome, which is only found in *A*. *glandulifera *[45].

The species of section *Arachis *were placed in two clades. The first one was divided into sub-clades. The first sub-clade comprised *A. hoehnei *(1,2,3), *A. stenosperma *(1,2), *A. kuhlmannii *(1,2*), A. helodes *and *A*. *linearifolia; *the second *A. cardenasii, A. kuhlmannii *(4), *A. simpsonii*, *A. monticola *and *A. duranensis *and the third comprised *A*. *hypogaea*, *A. correntina*, *A. microsperma*, *A. schininii*, *A. villosa *and *A. kuhlmannii *(3). *Arachis *sp (1) was very related to the A genome species but it was not included in any of the above clades. The second clade of section *Arachis *species was divided into two sub-clades. The first included 2n = 2x = 20 species and the other, species with 2n = 2x = 18 (*A. decora*, *A. praecox *and *A. palustris*). The species with 2n = 20 were separated into two sister subclades, one of them being formed only by B genome species (*A. magna*, *A. valida*, *A. ipaënsis *and *A*. *williamsii*) and the other included *A. batizocoi *(B genome), *A. cruziana *(B genome) and *A. glandulifera *(D genome). *Arachis batizocoi *and *A. cruziana *were very recently described as having a K genome based on FISH mapping of rDNA loci and heterochromatin detection [21].

The first clade of section *Arachis *included all the A genome species and *A. hoehnei*. It was believed that *A*. *hoehnei *did not present the small "A" chromosome pair [39]. *Arachis hoehnei *also grouped to A genome species based on the polymorphism of *trn*T-F region [18] and RAPD markers [27], but grouped to other B genome species based on microsatellite markers [13], and to the aneuploid (2n = 18) species with AFLP markers [18]. In our study, three different accessions of this species were included in the analysis and all of them were placed close to the A genome species, confirming that this species was correctly placed on the phylogenetic tree. The cytogenetical analysis of *A*. *hoehnei *was recently re-done and it was verified that this species has the A chromosome pair [21]. Thus, our data suggested A genome species are monophyletic.

The second clade comprised all species of section *Arachis *that do not have the A chromosome pair. This clade included the 2n = 20 species that are classified as B genome species and the ones that possess 2n = 18 chromosomes (*A. decora*, *A. palustris*, and *A. praecox*). The 2n = 2x = 18 species lack the small pair of chromosomes characteristic of the A genome species [46]. Lavia [47] suggested that *A*. *palustris *was derived from a species with × = 10 chromosomes and these species are phylogenetically related to the B genomes. Analyses based on AFLP [18] and microsatellite [48] data placed those 2n = 2x = 18 species closely to A genome species. However, microsatellite markers [13] and sequencing of the *trn*T-F region [18] and also our data showed those species are more closely related to B genome species. Thus, our study corroborates previous ones [13,18] and suggested these species originated from B genome species.

As mentioned above, the non A genome species from section *Arachis *with 2n = 20 chromosomes were placed in two clades. Molecular evidences based on markers such as RFLPs [42,49], RAPD [6,50], AFLP [17], and microsatellites [48] suggested that section *Arachis *diploids lacking the small A chromosome pair comprise a very diverse group, of which *A. ipaënsis*, *A. magna*, *A. williamsii*, and *A. valida*, closely linked in the present analysis, plus *A. gregoryi *[12] are those more closely associated to the B genome of *A. hypogaea*/*A. monticola*. Hybrids between *A. ipaënsis *and *A. magna *have shown 84% viable pollen [1]. Other kinds of evidences, such as molecular [6,28,43] and morphological [51] data, showed that these two species are closely related. Crossings between *A. williamsii *and *A. ipaënsis *resulted in hybrids with 66.9% of pollen stainability [52]. If the latter is the donor of the B genome to *A. hypogaea *[25], our data suggest that *A. ipaënsis*, *A. williamsii*, *A. magna *and *A. valida *could be used for the improvement of the B genome of cultivated peanut. This would increase the variability available for this purpose since a single accession of *A. ipaënsis *is available.

*Arachis batizocoi *was very close related to *A. cruziana*. *Arachis batizocoi *is considered a good genetic bridge to transfer genes to cultivated peanut [53,54]. If the phylogenetic relationships were correlated with the crossability, as observed among the A genome species [55,56], *A. cruziana *would have some crossability with *A. batizocoi *and could also be used as bridge for gene introgression in *A. hypogaea*. In fact, F_1 _hybrids between these two species had 36.4% of pollen viability (male fertility) and 0.3 I (univalents) and 9.9 II (bivalents) [18]. Thus, our data suggested that *A. cruziana *also can be a source of genes to *A. hypogaea *since it is very related to *A. batizocoi*.

*Arachis glandulifera *is classified as D genome since it does not cross with *A. hypogaea *and it has the most asymmetrical karyotype in genus *Arachis *[45]. *Arachis glandulifera*, like the B genome species, does not show the small pair of chromosomes found in the A genome species. Isoenzyme [10], RFLP [42], RAPD [6], AFLP [18] and cytogenetical [57] data also showed that *A. batizocoi *and *A. glandulifera *were closely related. Our data suggest that *A. glandulifera *may be derived from a B genome ancestor species.

Through the analysis of the heterochromatic bands and 45 S rDNA loci patterns, the species previously classified as B genome were arranged into three groups called B (*A. ipaënsis*, *A. magna*, *A. gregoryi*, *A. valida *and *A. williamsii*), K (*A. batizocoi*, *A. cruziana*, and *A. krapovickasii*) and F (*A. benensis *and *A. trinitensis*) [21]. Our data supported the classification in B and K genomes. We have not analyzed species of the new F genome.

### Sections *Trierectoides*, *Erectoides *and *Procumbentes*

As mentioned before the circumscriptions of these three sections were not clear and because of that their results were presented and discussed together. Our results agreed with the classification proposed by Krapovickas [3] which had all species from those three sections in only one section, called *Erectoides*. The data partially agreed also to a more recent classification proposed by the same author [5], that divided section *Erectoides *in two sections (*Erectoides *and *Procumbensae*), since as it can be seen in Figure 1, species of *Erectoides *and *Trierectoides *were all in a same sub-clade and species of *Procumbentes *were placed all together with *A. paraguariensis *and *A*. *glabrata*.

*Arachis paraguariensis *shows low genetic affinity to the other species of section *Erectoides *and because it shows some morphological peculiarities in the root, flowers and fruits it was suggested that this species should be segregated in an independent section [1]. Our results also raised doubt about the classification of this species, and suggested that *A. paraguariensis *might be classified as belonging to section *Procumbentes*. The segregation of *A. paraguariensis *from other *Erectoides *species was also observed in phylogeny based on ITS data using parsimony analysis [22].

The placement of *A. glabrata *in a clade with *Procumbentes *and *Erectoides *species will be discussed in the section *Rhizomatosae *item.

The clade formed by sections *Erectoides*, *Trierectoides *and *Procumbentes *was the most related to section *Arachis*. Crossability data between members of sections *Arachis *and *Erectoides *suggest that these two sections are more phylogenetically related to each other than to the other sections of the genus since pollination has lead to fertilization. Although there has been no development of the resulting proembryos [58], members of the other sections do not even show such a degree of success on crossings with section *Arachis *[55]. Isozyme and protein pattern data [8,10] suggested that section *Erectoides *and *Procumbentes *are closely related to the section *Arachis*.

### Section *Caulorrhizae*

The morphological traits that have been traditionally used to distinguish the two type specimens of *A. pintoi *(GK 12787) and *A. repens *(GKP10538) are not sufficient to differentiate all the accessions collected, as they show intermediate phenotypes between the extreme types [59].

The clade was formed by the two species of section *Caulorrhizae*, confirming that *A. pintoi *and *A. repens *are very closely related. There were no differences between the sequences of *A. pintoi *(CIAT 22237 = W132) and *A. repens *(V 5868), and few differences of these two in relation to accession V 6791, also considered as *A. pintoi*. RAPD data also suggested they are very closely related [37]. F_1 _hybrids between accession GK 12787 of *A. pintoi *and accession GKP 10538 of *A. repens*, which represent the extreme types, had 86.8% pollen fertility [55], which is higher than the level of pollen fertility found in intraespecific hybrids of crosses between accessions of some other *Arachis *species [45,60].

### Section *Triseminatae*

The phylogenetic data showed that *A. triseminata *was not closely related to species of any other sections, in agreement with its placement in a separate section (*Triseminatae*) [1]. No successful crossings among *A*. *triseminata *and members of other sections were obtained [1,55], showing its genetic isolation from other sections of genus *Arachis*.

### Section *Heteranthae*

*Arachis pusilla*, the only species of section *Heteranthae *included in this study, had a basal position, on the first radiation of genus *Arachis *(Figure 1) and closely related to *Extranervosae*, which formed the second radiation in the genus. The affinity of *Heteranthae *and *Extranervosae *sections agreed with floral morphology, geographical distribution and crossability data, since the only known intersectional hybrid of *Extranervosae *was obtained from a cross with *Heteranthae *[1].

### Section *Rhizomatosae*

Cytogenetic data suggested that the origins of *Arachis *tetraploid species (*A. glabrata *and *A. hypogaea*) were independent [61]. An analysis using RFLP markers also showed that *A. glabrata *was very distinct from *A. hypogaea *[49]. In the present study, *A. glabrata *and *A. hypogaea *were placed in different clades, confirming that the polyploid species evolved independently in the genus.

*Arachis glabrata *was more related to species of section *Procumbentes *than to *A. burkartii *which is also traditionally allocated in section *Rhizomatosae*, although in a series of its own. These species differed on the ploidy level, since *A. glabrata *is a tetraploid (2n = 4x = 40) and *A*. *burkartii *a diploid species. Crossings between *A. glabrata *and diploid species from other sections resulted in hybrids, in contrast to *A. burkartii*, for which no hybrids were obtained from numerous attempted crosses [55]. The other tetraploid species analyzed (*A. hypogaea*) was placed close to diploid species that have similar genomes and are certainly involved in its origin [1,25]. Thus, the data indicated that the referred species of section *Rhizomatosae *did not have a monophyletic origin.

*Arachis glabrata *was placed in a clade with species from section *Erectoides *and *Procumbentes*. Tetraploid species of section *Rhizomatosae *were classified as EERR [55], and at that time the EE crossing group was attributed to section *Erectoides*, which comprised all species that, according to the last classification [1], are distributed in sections *Procumbentes*, *Erectoides *and *Trierectoides*. Thus, based in our and previous data we suggested the following hypothesis to the origin of *A. glabrata*: 1) *A. glabrata *originated from species of the *Erectoides *group, with rhizomes appearing twice, independently, in the evolution of genus *Arachis*; 2) *A. glabrata *is an allopolyploid EERR, as previously suggested [55], that resulted from a cross between one species from section *Erectoides *and one species from section *Rhizomatosae*, that had a genome not similar to the one found in *A. burkartii*.

## Conclusion

The main clades established in this study in general agreed with many other studies that have used other types of evidences (morphological, crossability, biochemical, cytogenetical and molecular) and different species, being some of them included in our study and some not. Thus, the relationships established do reflects the affinity of the species, and that can be a useful framework for future systematic reviews of genus *Arachis *and for the selection of species to pre-breeding programs.

## Methods

### Plant material

A total of 55 accessions, which represent 45 species and the nine sections of the genus *Arachis *were analyzed (Table 2). These accessions were obtained from the Brazilian *Arachis *Germplasm Collection, maintained at Embrapa Recursos Genéticos e Biotecnologia - CENARGEN (Brasília-DF, Brazil). All plants were grown from seed or cuttings (sections *Rhizomatosae *and *Caulorrhizae*) under greenhouse conditions prior to DNA extraction. *Stylosanthes capitata*, *S*. *hamata*, *S*. *viscosa*, *Chapmannia gracilis *and *C. sericea *were used as outgroups, because these genera are considered to be closely related to *Arachis *[1,30,31,62].

**Table 2:** Accessions of genus Arachis analyzed in this study.

Species	Accession	Genome ^1^	Chromosome number/ Ploidy level ^2^	GenBank Accession Numbers
**Sect. *Arachis***				
***A. cardenasii ***Krapov. & W.C.Greg.	GKP 10017	A	2n = 2x = 20	AY615236
***A. correntina ***(Burkart) Krapov. & W.C.Greg.	CIAT 22249	A	2n = 2x = 20	AF203554
***A. duranensis*** Krapov. & W.C.Greg.	VNvEc 14167	A	2n = 2x = 20	AY615240
***A. helodes*** Mart. ex Krapov. & Rigoni	VSGr 6325	A	2n = 2x = 20	AY615241
***A. kuhlmannii*** (1) Krapov. & W.C.Greg.	VPoBi 9375	A	2n = 2x = 20	AY615232
***A. kuhlmannii*** (2)	VSGr 6404	A	2n = 2x = 20	AY615219
***A. kuhlmannii*** (3)	VPzRcSgSv 13530	A	2n = 2x = 20	AY615238
***A. kuhlmannii ***(4)	VSPmSv 13721	A	2n = 2x = 20	AY615243
***A***. sp (1)	VSPmSv 13736	A	2n = 2x = 20	AY615226
***A. linearifolia ***Valls, Krapov. & C.E.Simpson	VPoBi 9401	A	2n = 2x = 20	AY615242
***A. microsperma ***Krapov., W.C.Greg. & Valls	VRGeSv 7681	A	2n = 2x = 20	AY615221
***A. schininii ***Krapov., Valls & C.E.Simpson	VSW 9923	A	2n = 2x = 20	AY615248
***A. simpsonii*** Krapov. & W.C.Greg.	VSPmSv 13728	A	2n = 2x = 20	AY615247
***A. stenosperma ***(1) Krapov. & W.C.Greg.	Lm 1	A	2n = 2x = 20	AY615252
***A. stenosperma ***(2)	VSPmW 13844	A	2n = 2x = 20	AY615227
***A. villosa ***Benth.	VGoMrOv 12812	A	2n = 2x = 20	AY615215
***A. hypogaea*** L.	Mf 1560	AB	2n = 4x = 40	AY615267
***A. monticola*** Krapov. & Rigoni	VOa 14165	AB	2n = 4x = 40	AY615239
***A. batizocoi*** Krapov. & W.C.Greg.	K 9484	B	2n = 2x = 20	AY615256
***A. cruziana*** Krapov., W.C.Greg. & C.E.Simpson	WiSVg 1302	B	2n = 2x = 20	AY615259
***A. hoehnei ***(1) Krapov. & W.C.Greg.	KG 30006	A	2n = 2x = 20	AY615223
***A. hoehnei ***(2) Krapov. & W.C.Greg.	VPoBi 9146	A	2n = 2x = 20	AY615224
***A. hoehnei*** (3) Krapov. & W.C.Greg.	VPoBi 9140	A	2n = 2x = 20	AY615222
***A. ipaënsis*** Krapov. & W.C.Greg.	KGBPSSc 30076	B	2n = 2x = 20	AY615257
****A*. *magna ****(1) Krapov., W.C.Greg. & C.E.Simpson	KGSSc 30097	B	2n = 2x = 20	AY615230
***A. magna*** (2)	VSPmSv 13760	B	2n = 2x = 20	AY615231
***A. williamsii*** Krapov. & W.C.Greg.	WiDc 1118	B	2n = 2x = 20	AY615255
***A. valida ***Krapov., & W.C.Greg.	VPoBi 9153	B	2n = 2x = 20	AY615244
***A. glandulifera*** Stalker	VSPmSv 13738	D	2n = 2x = 20	AY615258
***A. decora*** Krapov., W.C.Greg. & Valls	VSPmPzRs 13290	Unknown	2n = 2x = 18	AY615237
***A. palustris*** Krapov., W.C.Greg. & Valls	VPmSv 13023	Unknown	2n = 2x = 18	AY615238
***A. praecox ***Krapov., W.C.Greg. & Valls	VSGr 6416	Unknown	2n = 2x = 18	AY615234
				
**Sect. *Erectoides ***Krapov. & W.C.Greg.				
***A. brevipetiolata ***Krapov. & W.C.Greg.	VMPzW 13959	E	2n = 2x = 20	AY615251
***A. hermannii ***Krapov. & W.C.Greg.	VPoJSv 10390	E	2n = 2x = 20	AY615260
***A. major ***(1) Krapov. & W.C.Greg.	VRGeSv 7644	E	2n = 2x = 20	AY615229
***A. major*** (2)	VRGeSv 7632	E	2n = 2x = 20	AY615228
***A. paraguariensis*** Chodat & Hassl. (1) subsp. *capibarensis *Krapov. & W.C.Greg.	VMPzW 14024	E	2n = 2x = 20	AY615217
***A. paraguariensis*** (2) subsp. *paraguariensis *Chodat & Hassl.	VRGeSv 7677	E	2n = 2x = 20	AY615218
				
**Sect. *Trierectoides ***Krapov. & W.C.Greg.				
***A. guaranitica*** Chodat & Hassl.	VRcSgSv 13600	E	2n = 2x = 20	AY615261
***A. tuberosa*** Bong. ex Benth	VRGeSv 7607	E	2n = 2x = 20	AY615235
				
**Sect. *Procumbentes ***Krapov. & W.C.Greg.				
***A. appressipila*** Krapov. & W.C.Greg.	GKP 10002	E	2n = 2x = 20	AY615254
***A. kretschmeri*** Krapov. & W.C.Greg.	KrRy s/n (IRFL 2273)	E	2n = 2x = 20	AY615220
***A. matiensis*** Krapov., W.C.Greg. & C.E.Simpson	VSPmSv 13718	E	2n = 2x = 20	AY615249
***A. pflugeae*** C.E.Simpson, Krapov. & Valls	VRcSgSv 13589	E	2n = 2x = 20	AY615233
***A.*** sp (2)	VMPzW 14044	E	2n = 2x = 20	AY615225
				
**Sect. *Rhizomatosae ***Krapov. & W.C.Greg.				
**Ser. *Prorhizomatosae ***Krapov. & W.C.Greg.				
***A. burkartii*** Handro	VZnMrOvW 12322	R	2n = 2x = 20	AY615245
**Ser. *Rhizomatosae***				
***A. glabrata*** var. *glabrata *Benth.	Cv. Florigraze	R	2n = 4x = 40	AY615250
				
**Sect. *Caulorrhizae ***Krapov. & W.C.Greg.				
***A. pintoi*** Krapov. & W.C.Greg. (1)	VSWSa 6791	C	2n = 2x = 20	AY615263
***A. pintoi*** (2)	CIAT 22237 = W132	C	2n = 2x = 20	AF203551
***A. repens*** Handro	V 5868	C	2n = 2x = 20	AY615264
				
**Sect. *Triseminatae ***Krapov. & W.C.Greg.				
***A. triseminata*** Krapov. & W.C.Greg.	W 195	T	2n = 2x = 20	AY615253
				
**Sect. *Heteranthae ***Krapov. & W.C.Greg.				
***A. pusilla*** Benth.	VRSv 10833	AM	2n = 2x = 20	AY615216
				
**Sect. *Extranervosae ***Krapov. & W.C.Greg.				
***A. burchellii*** Krapov. & W.C.Greg.	VGaRoSv 12618	EX	2n = 2x = 20	AY615262
***A. lutescens ***Krapov. & Rigoni	VSStGdW 7741	EX	2n = 2x = 20	AY615246
***A. villosulicarpa*** Hoehne	VKSSv 8816	EX	2n = 2x = 20	AY615265
				
**Outgroups**				
***Chapmannia gracilis*** Balf.f Thullin	Miller & Alexander14039(E)	Not described in the source	Not described in the source	AF203545
***Chapmannia sericea:*** Thulin & Mc Kean	Miller & Alexander14241(E)	Not described in the source	Not described in the source	AF203548
***Stylosanthes capitata*** Vogel	CIAT1693	Not described in the source	2n = 4x (40)	AF203549
***Stylosanthes hamata***	Beyra M 595 (MONT)	Not described in the source	2n = 2x(20)	AF203550
***Stylosanthes viscosa*** Sw.	Clemente J C	Not described	2n = 2x(20)	AY6152141

Collectors: B = D.J.Banks; Bi = L.B.Bianchetti; Dc = D. Claure; Ec = E.D.Cruz; G = W.C.Gregory; Ga = M.L.Galgaro; Gd = I.J.Godoy; Ge = M.A.N.Gerin; Go = K.E.Gomes; Gr = A.Gripp; J = L.Jank; K = A.Krapovickas; Kr = A.Kretschmer Jr.; Lm = L.Monçato; M = J.P.Moss; Mf = Est.Exp.Agr. Manfredi, Córdoba, Argentina; Mr = C.O.C.Moraes; Nv = L.Novara; Oa = O.Ahumada; Ov = J.C.Oliveira; P = J.Pietrarelli; Pm = R.N.Pittman; Po = A.Pott; Pz = E.A.Pizarro; R = V.R.Rao; Rc = R.C.Oliveira; Ro = D.M.S.Rocha; Rs = R.C.Santos; Ry = P.R.Rayman; S = C.E.Simpson; Sa = J.M.Santos; Sc = A. Schinini; Sg = A.K.Singh; St = H.T.Stalker; Sv = G.P.Silva; V = J.F.M.Valls; Vg = I.Vargas; W = W.L.Werneck; Wi = D.E.Williams; Zn = A.Zanin

1 - Genome designations follow abreviations proposed by Smartt & Stalker [61] for taxonomic sections and group of species in section *Arachis *[53].

2 - Chromosome numbers are the observed for each species and compiled by Fernández & Krapovickas [39], Lavia & Fernández [44], Lavia *et al *[46,70]. Eventually, counts were obtained by the accessions listed.

### DNA extraction and PCR amplification

DNA was extracted from young leaflets of single plants, using a procedure previously described [63]. Primers **ITS5 **(5'GGAAGTAAAAGTCGTAACAAGG3') and **ITS4 **(5'TCCTCCGCTTATTGATATGC3') were used to amplify the two internal transcribed regions, ITS1 and ITS2, and the 5.8 S gene [64]. Each amplification reaction contained 12 μl of water, 1.5 μl of magnesium chloride (50 mM), 2.6 μl of 10× *Taq *polymerase reaction buffer, 1.5 μl of each primer (10 mM), 5.0 μl of a 5 ng/μl DNA dilution, 2.2 μl of dNTPs (2.5 μM each) and 0.2 μl of *Taq *DNA polymerase (5 U/μl). The reactions were performed on a PTC 100 (MJ Research) using the following program: an initial denaturing step (2 min at 94°C) followed by 35 cycles of the following steps: denaturing (1 min at 95°C), annealing (1 min at 55°C) and extension (1.30 min at 72°C); and a final extension step of 10 min at 72°C. The PCR products were purified using the kit Concert™Rapid PCR Purification System (Life Technologies) before sequencing.

### DNA sequencing

PCR products were sequenced using the procedure proposed by Sanger *et al*. [65]. Each sequence reaction contained: 2 μl of Big Dye™Terminator (Applied Biosystems), 1.5 μl of PCR product (5 ng/μl), 0.5 μl of primer solution (0.25 mM) and water up to 10 μl. The primers used on the sequencing reactions were the same used on the amplification of the target fragments. The sequencing reactions were performed on a PTC 100 (M.J. Research) using the following program: 1 min at 96°C, 40 cycles of 10 sec at 96°C; 10 sec at 55°C; 4 min at 60°C. The sequencing was performed in an ABI PRISM 377 Automated DNA Sequencer (Perkin-Elmer/Applied Biosystems). Each DNA strand was sequenced at least twice to ensure the accuracy of the results.

### Sequence and phylogenetic analysis

The phylogenetic analysis was the Bayesian Inference with the MCMC calculations implemented by Mr. Bayes 3.1 [66]. The best evolution model (GTR+G) was selected using mrmodeltest [67] and PAUP 4b10 [68]. The model parameters were set in the alignment nexus file and then ran in Mr. Bayes 3.1, which performed 20,000,000 of generations, sampling trees in each 100 generations. The first 1250 trees were eliminated as the burn-in. The 50% majority consesus trees was inspected and prepared in figtree software.

## Authors' contributions

All authors read and approved the final manuscript. MDB carried out the data collection and analysis and drafted the manuscript. MCM participated in the drafting of the manuscript. DAP and JPM participated in the sequencing and sequence analysis. MBJ and JMJ participated in the phylogenetic analysis. JFMV participated in the conception of the project and provided the germplasm. CRL and MAG participated in conceiving the study and analysis, and participated in drafting the manuscript.
